# Comparison of the sensitivity of mammography, ultrasound, magnetic resonance imaging and combinations of these imaging modalities for the detection of small (≤2 cm) breast cancer

**DOI:** 10.1097/MD.0000000000026531

**Published:** 2021-07-02

**Authors:** Hai-long Chen, Jiao-qun Zhou, Qiang Chen, Yong-chuan Deng

**Affiliations:** a Department of Breast Surgery, the Second Affiliated Hospital of Zhejiang University School of Medicine; b Department of Surgical oncology, the First People's Hospital of Fuyang Hangzhou, Zhejiang Province, China.

**Keywords:** magnetic resonance imaging, mammography, sensitivity, small breast cancer, ultrasound

## Abstract

The aim of this study was to compare the sensitivity of mammography (MG), ultrasound (US), magnetic resonance imaging (MRI), and combinations of these imaging modalities for the detection of small (≤2 cm) breast cancer and to evaluate the benefit of preoperative breast MRI after performing conventional imaging techniques for small breast cancer.

This was an observational retrospective review of 475 patients with pathologically confirmed breast cancer. We reviewed the medical records; assessed the preoperative reports of MG, US, and MRI; and categorized them as benign features (BI-RADS 1–3) or malignant features (BI-RADS 4 or 5). The criterion standard for detection was the pathologic assessment of the surgical specimen. The sensitivities of the different techniques were compared using the McNemar test.

Among the 475 women, the sensitivity of MG was significantly greater in patients with low breast density than in those with high breast density (84.5% vs 65.8%, *P* < .001). US had higher sensitivity than MG (*P* < .001), and the combination of MG + US showed better sensitivity than MG or US alone (*P* < .001). Further addition of MRI to the combination of MG and US statistically contributed to the sensitivity yield (from 93.3% to 98.2%; *P* < .001) but did not significantly increase the mastectomy rate (from 48.2% to 49.3%; *P* = .177).

MG has limited diagnostic sensitivity in patients with small breast cancer, especially in those with dense breast tissue. US is better than MG at detecting small breast cancer, regardless of breast density. The addition of MRI to MG and US could increase sensitivity without increasing the mastectomy rate. This study suggests performing MRI routinely on the basis of MG and US for small (≤2 cm) breast cancer.

## 1 Introduction

Breast cancer is the most commonly diagnosed cancer and the leading cause of cancer-related deaths among women worldwide.^[1]^ In breast cancer, like many other cancers, the stage of disease when diagnosed is significantly correlated with prognosis, and early identification and diagnosis of breast cancer greatly increases the likelihood of curing the disease. Tumor size plays an important role in the breast cancer stage, and a smaller tumor size indicates a lower rate of axillary lymph node metastasis and a better prognosis. Therefore, the identification of small malignant breast masses is particularly important in the treatment of breast cancer.

With the development and improvement of imaging technologies, mammography (MG) has significant value for the early detection of breast cancer, it is the screening test that has been proven to reduce breast cancer mortality^[2,3]^ and is often regarded as the preferred method. MG can detect malignant calcifications, including ductal carcinoma in situ (DCIS). However, MG is not a perfect test, and its overall sensitivity is 75% to 85%, which decreases sharply for dense breast parenchyma,^[4,5]^ for small breast cancer, its diagnostic efficiency is still disputable.

As a widely available tool, ultrasound (US) can overcome the limitations of MG in some patients. US has also been used extensively for the screening and diagnosis of breast cancer, with a high sensitivity of 76% and specificity of 84%.^[6]^ Screening with US increases the breast cancer detection rate in women at average risk.^[7,8]^ Additionally, breast magnetic resonance imaging (MRI) can be a valuable supplement to MG and US. It has been reported in several studies that MRI provides considerable increased detection in high-risk women than combination of US and MG screening,^[9–11]^ but the use of MRI remains controversial because of the disadvantage of more false-positive results, which lead to unnecessary mastectomies.

However, the tumor size may affect the sensitivity of different techniques; studies on small breast cancer (maximum diameter ≤2.0 cm) are limited. In this study, we analyzed the diagnostic value of US and MG in small breast cancer (≤2.0 cm) and evaluated the value of MRI in these breast cancer patients.

## 2 Methods

### 2.1 Patients

Patients histologically confirmed breast cancer at the Second Affiliated Hospital of Zhejiang University from January 1, 2015 to June 30, 2019 were identified through the electronic medical records. Women were included in this study if they met the following criteria: mastectomy or lumpectomy for breast cancer were performed; MG and US were performed within the 1-month period before core needle biopsy or surgical excision for breast lesions; pathologically confirmed invasive breast cancer or DCIS; pathologically confirmed the maximum diameter of the tumors ≤2.0 cm; no metastatic disease and no neoadjuvant therapy. A total of four hundred and seventy-five patients were included in this observational retrospective study, there personal data, diagnostic process, surgical type, and pathology results were collected. The dates of data collection ranged from November 4, 2019 to January 15, 2020.

This study was approved by the Ethics Committee of the Second Affiliated Hospital of Zhejiang University School of Medicine. The need for informed consent was waived by the Ethics Committee because the study was an observational, retrospective study; the patients’ identification information had been removed.

### 2.2 Clinical imaging

All patients underwent routine preoperative MG and US. MG (2-view) was performed using a Hologic unit (Selenia, Hologic, Santiago, USA) in 2 standard imaging planes (standard 45 degree mediolateral oblique and craniocaudal). The mammographic density was estimated from the mammograms according to the American College of Radiology (ACR) guidelines: a = fatty, b = scattered fibroglandular, c = heterogeneously dense, and d = extremely dense. ACR a and b were indicated as low density and ACR c and d as high density. US was performed using high-quality equipment (IU Elite; Philips Healthcare, Best, Netherlands). Patients were fully exposed in a supine position with their arms raised above their head. A 6 to 12 MHz high-frequency probe was used to scan the bilateral breasts and axillaries of the patients. MRI examination was performed using a 3.0 T system (Signa HDx, GE Healthcare, USA) witha 8-channel breast coil. Core sequences include axial STIR, 3D axial T1, T2 sagittal fat saturated, axial DWI, 3D T1-weighted volume imaging for breast assessment (VIBRANT) dynamic gradient-echo sequence, with an injection of gadopentetate dimeglumine as the contrast agent. Patients were in a prone position for the MRI examination.

MG, US, and MRI images were categorized according to the BI-RADS score. For the analyses, those cases classified as BI-RADS categories 4 and 5 were considered as malignant features, whereas categories 1, 2, and 3 were considered as benign features.

### 2.3 Pathologic assessment

All patients underwent mastectomy or lumpectomy for breast cancer. The surgical specimens were assessed with hematoxylin-eosin staining, and an immunohistochemical study was performed. Tumor size was obtained from pathology reports. In patients with multifocality/multicentricity tumors, tumor size was determined with the diameter of the largest tumor focus. Estrogen receptor (ER) positivity and progesterone receptor (PR) positivity were considered the presence of ≥1% nuclear-stained malignant cells. ER-positive or PR-positive were designated as hormone receptor (HR) positive, whereas ER-negative and PR-negative were designated as HR-negative. The status of human epidermal growth factor receptor 2 (HER2) was based on a semiquantitative method of calculating the intensity of nuclear staining in tumor cells, which was graded between 0 and 3+ (with higher scores indicating higher staining intensity). Results of “0,” “1+,” and “2+” which designated HER2-FISH negative were reported as HER2 negative; for positive status, “3+” and “2+” with HER2-FISH positive were reported.

### 2.4 Statistics

This study was an exploratory analysis of observational data collected in routine clinical practice. Using a *χ*^2^ test, patient characteristics and clinical features were compared between the benign features group and malignant features group based on the BI-RADS classification. The McNemar test was used to compare the sensitivity of the different techniques across the whole sample group. The same test was used to compare the mastectomy rate with and without MRI. Data analysis was performed using SPSS version 16.0 software (SPSS Inc, Chicago, IL). A *P* value of <.05 was considered statistically significant.

## 3 Results

A total of 475 women were included in our study, and the raw data were shown in Supplemental Digital Content file 1. The clinicopathological features of patients were shown in Table 1, the median patient age was 51 years (range, 27–89 years). A total of 92 (19.4%) patients were diagnosed with DCIS. There were 254 (53.5%) patients in the group with tumors sized 1.1 to 2.0 cm, and 221 (46.5%) patients were in the tumor size ≤1.0 cm group, 14 of whom had tumors ≤0.5 cm. A total of 228 (48.0%) patients were 50 years or younger, and 178 (78.1%) of these patients had a high breast density, which was higher than that of patients older than 50 years (78.1% vs 52.2%, *P* < .001). The sensitivity of MG was significantly greater in patients >50 years (81.3% vs 62.7%, *P* < .001), patients with low breast density (84.5% vs 65.8%, *P* < .001), patients with tumor size >1.0 cm (78.7% vs 65.2%, *P* = .001), and patients with positive lymph nodes (85.7% vs 69.6%, *P* = .003). However, there was no significant difference in sensitivity of US among age groups (*P* = .790) or breast density groups (*P* = .526), but sensitivity increased significantly in patients with larger tumor sizes (92.9% vs 83.7%, *P* = .002) and patients with positive lymph nodes (97.6% vs 86.7%, *P* = .001). Moreover, patients with DCIS had significantly lower sensitivity according to US than those with invasive carcinoma (78.3% vs 91.1%, *P* < .001). There were no significant differences among the different molecular subtypes with MG or US.

**Table 1:** Clinicopathological features of patients.

	Mammography (n = 475)			Ultrasound (n = 475)			MRI (n = 282)		
	Mal	Ben	*P*	Mal	Ben	*P*	Mal	Ben	*P*
Age									
≤50	143	85	**<.001**	203	25	.790	137	9	.712
>50	201	46		218	29		129	7	
Breast density									
Low (ACR a/b)	142	26	**<.001**	151	17	.526	93	7	.475
High (ACR c/d)	202	105		270	37		173	9	
Tumor size									
≤1.0 cm	144	77	**.001**	185	36	**.002**	122	12	**.021**
1.1–2.0 cm	200	54		236	18		144	4	
Lymph nodes									
N0	272	119	**.003**	339	52	**.001**	203	16	**.004**
N1–3	72	12		82	2		63	0	
Histological type									
	64	28	.495	72	20	**<.001**	39	9	**<.001**
Invasive	280	103		349	34		227	7	
HR/HER2 status									
HR+/HER2−	205	78	.069	250	33	.676	170	7	**.005**
HR+/HER2+	57	12		64	5		36	3	
HR−/HER2+	39	24		55	8		24	6	
HR−/HER2-	43	17		52	8		36	0	

ACR = American College of Radiology, Ben = benign features (BI-RADS 1–3), DCIS = ductal carcinoma in situ, HER2 = human epidermal growth factor receptor 2, HR = hormone receptor, Mal = malignant features (BI-RADS 4,5). Bold type represents statistically significance (*P* < .05).

**Figure 1:**
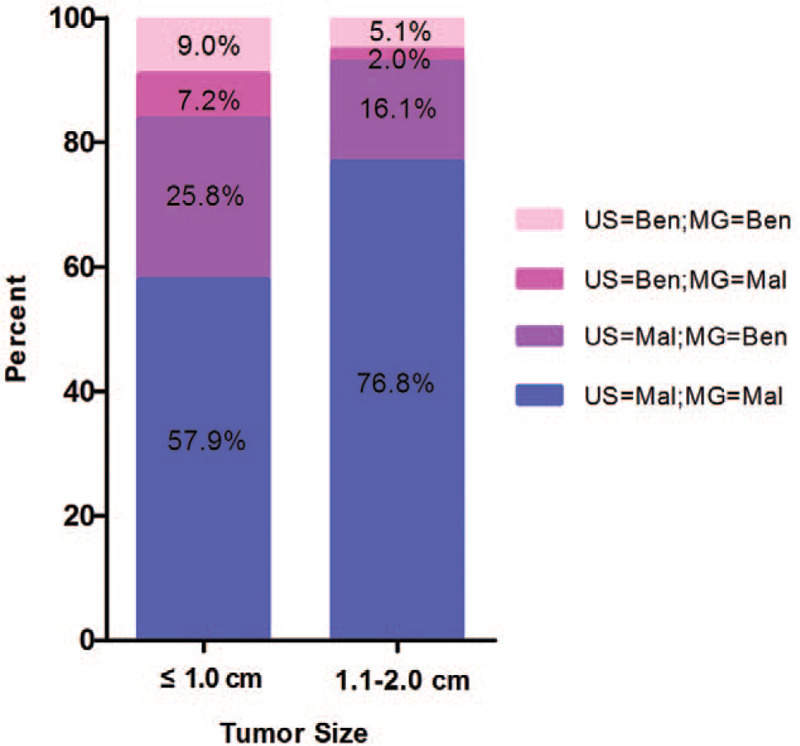
Stacked bar plots displaying BI-RADS classification (benign and malignant) for ultrasound (US) and mammogram (MG) based on the tumor size. Percent is displayed in the bars.

For the whole sample group, US had higher sensitivity than MG (*P* < .001), and the combination of MG + US exhibited better sensitivity than MG or US alone and showed significant differences (*P* < .001) (Table 2; Fig. 1). However, there was no significant difference between MG+US vs US in the 1.1 to 2.0 cm group (94.9% vs 92.9%; *P* = .063) (Table 2).

**Table 2:** Comparison of the sensitivities of the different techniques in the whole sample group (McNemar test).

	≤1.0 cm (n = 221)	1.1–2.0 cm (n = 254)	Total (n = 475)
MG	144 (65.2%)	200 (78.7%)	344 (72.4%)
US	185 (85.1%)	236 (92.9%)	421 (88.6%)
MG + US	201 (91.0%)	241 (94.9%)	442 (93.1%)
*P*			
MG vs US	**<.001**	**<.001**	**<.001**
MG vs MG + US	**<.001**	**<.001**	**<.001**
US vs MG + US	**<.001**	.063	**<.001**

MG = mammography, US = ultrasound. Bold type represents statistically significance (*P* < .05).

Among the 475 patients, 282 underwent breast MRI within 1-month before pathologically diagnosis and the remaining 193 patients did not. The sensitivity of MRI increased significantly in patients with larger tumor sizes (97.3% vs 91.0%, *P* = .021), patients with positive lymph nodes (100.0% vs 92.7%, *P* = .004). Besides, it showed greater sensitivity in patients with triple-negative breast cancer (Table 1).

There were no differences in histological type (*P* = .118), tumor size (*P* = .601) and lesion number (*P* = .487) between the MRI and without MRI groups. A total of 83 (48.2%) patients underwent mastectomies in the no MRI group, and the number of mastectomies was 139 (49.3%) in the MRI group. Thus, in our study, the use of MRI did not significantly increase the mastectomy rate (from 48.2% to 49.3%; *P* = .177) (Table 3).

**Table 3:** Pathological features and surgery types of patients the with and without MRI groups.

	With MRI (n = 282)	Without MRI (n = 193)	*P*
Histological type			
DCIS	48 (17.0%)	44 (22.8%)	.118
Invasive	234 (83.0%)	149 (77.2%)	
Tumor size			
≤1.0 cm	134 (47.5%)	87 (45.1%)	.601
1.1–2.0 cm	148 (52.5%)	106 (54.9%)	
Lesions			
Unifocal	263 (93.3%)	183 (94.8%)	.487
MF/MC	19 (6.7%)	10 (5.2%)	
Surgery			
Wide local excision	143 (50.7%)	110 (51.8%)	.177
Mastectomy	139 (49.3%)	83 (48.2%)	

DCIS = ductal carcinoma in situ, MC = multicentric, MF = multifocal, MRI = magnetic resonance imaging.

Out of the 282 tumors, the combination of MG and US detected 263 (93.3%) and the addition of MRI detected 277 (98.2%). Furthermore, the addition of MRI to the combination of MG and US statistically contributed to the increased sensitivity (*P* < .001), and the gain in sensitivity was observed in both tumor size groups (Table 4).

**Table 4:** Comparison of the sensitivities of MG + US and MG + US + MRI with respect to tumor size (McNemar test).

	MG + US	MG + US + MRI	*P*
≤1.0 cm (n = 134)	126 (94.0%)	132 (98.5%)	**.031**
1.1–2.0 cm (n = 148)	137 (92.6%)	145 (98.0%)	**.008**
Total (n = 282)	263 (93.3%)	277 (98.2%)	**<.001**

MG = mammography, MRI = magnetic resonance imaging, US = ultrasound. Bold type represents statistically significance (*P* < .05).

## 4 Discussion

In recent years, as its incidence has gradually increased, breast cancer has become the leading cancer affecting female health. Because of the diverse clinical features of early breast cancer, especially small tumors, the misdiagnosis and missed diagnosis rates are high. Therefore, early detection of small breast cancer is crucial to improve prognosis.

At present, MG is the preferred noninvasive modality for breast cancer screening because of its accessibility and availability. However, the sensitivity of MG is greatly affected by the density of breast tissue; thus, it can range from as high as 80% to 98% in women with fatty breast tissue to as low as 30% to 48% in women with dense breast tissue.^[12,13]^ Previous studies have reported that breast density is high in approximately 74% of women between 40 and 49 years of age and in 57% of women in their 50s.^[14]^ In our study, 78.1% of patients younger than 50 years and 52.2% of patients older than 50 years had high breast density, consistent with previous reports. In these patients with dense breast tissue, the sensitivity of MG dropped to 65.8%. Up to 80.2% (105/131) of cancers misdiagnosed using MG were located in dense breasts in our study. Our results show that MG alone is insufficient in small breast cancer, especially for women with dense breast tissue.

US is another imaging modality that is widely available and well tolerated by patients. The sensitivity of US is not affected by breast density. Screening US in women with dense breasts and negative MG can yield an incremental increase in the cancer detection rate of 3.7 to 4.2 more cancers identified per 1000 women screened.^[6,7,15]^ Asian women, compared with Western women, more often have small, dense breasts,^[16]^ and US is an appropriate tool for breast cancer detection in these women.^[17]^ In our study, 421 (88.6%) small cancers were detected by US, which was higher than MG, and we demonstrated that US was significantly better than MG at detecting malignancy in patients with high breast density. Although previous studies have shown that US has a lower sensitivity for in situ disease than MG,^[18,19]^ our results did not find a statistically significant difference between US and MG for the detection of in situ disease (78.2% vs 65.6%, *P* = .169). The possible reason for this finding is that MG does not have pronounced sensitivity for small breast cancer, and a high proportion of dense breast tissue may also contribute to this result. Overall, the results showed that US has an advantage over MG for detection of small breast cancer.

Meanwhile, we observed high sensitivity using US combined with MG for small (≤2.0 cm) breast cancer (93.1%). The combination of US and MG was significantly more sensitive than either modality used alone, with the exception of US in the 1.1 to 2.0 cm group, which did not reach the significance level (*P* = .063), this showed the superiority of US in the tumor size range 1.1 to 2.0 cm. However, we still found a notable efficiency in the detection of small breast cancer with the combination of US and MG.

Numerous studies have proven that MRI is the most sensitive modality for the detection of breast malignancies, with an estimated sensitivity of 95%,^[20–22]^ and some studies have reported that the addition of MRI to conventional techniques increases the sensitivity.^[20]^ However, in another study, 200 patients with breast cancer were retrospectively reviewed, and no significant increase in sensitivity by adding MRI to other conventional techniques was found.^[23]^ In our study, the total sensitivity of MRI alone was 94.7%, which is consistent with previous reports. We observed high MRI sensitivity for triple-negative breast cancer, previous study reported triple-negative breast cancer have characteristics in MRI, including a larger solitary lesion, mass with smooth mass margin, high signal intensity on T2-weighted images and rim enhancement,^[24]^ which is probably related with high sensitivity. However, the number of patients in our study is low, further work about triple-negative breast cancer and MRI is necessary.

Our study showed that the addition of MRI to US and MG resulted in a significant improvement in sensitivity (from 93.3% to 98.2%, *P* < .001), and an improvement was found in the two tumor size groups. These results demonstrated that the addition of MRI to US and MG is beneficial for detecting small breast cancer.

Nowadays, the use of preoperative breast MRI is still controversial for some reasons such as the increase in the mastectomy rate.^[25,26]^ However, our data show that the use of MRI did not significantly increase the mastectomy rate. The main reason was detection of multifocality/multicentricity of breast cancer, MRI sensitivity for multifocality/multicentricity is greater compared with mammography.^[27]^ It was reported that advanced pT and pN stages were closely associated with the presence of multifocality/multicentricity tumors.^[28]^ In our study, multifocality/multicentricity was only found in 6.1% (29/475) patients, possibly because the tumor size of patients was small. The probability of detection of extra lesions by additional MRI that could change the management was low. Therefore, MRI did not increase the mastectomy rate in small breast cancer. However, the mastectomy rate is also influenced by the subjective willingness of patients and the use of oncoplastic techniques.

Our study is limited because we only included patients diagnosed with breast cancer, the sensitivities of the imaging modalities were assessed, but the specificities could not be achieved. Therefore, this study can help us to reduce the rate of missed diagnosis, but the rate of misdiagnosis is unknown. Furthermore, the results of this study also cannot be applied to a population-based screening due to the design. Besides, the number of patients with tumors ≤0.5 cm was too few to interpret data for that group. Another limitation is that we retrospectively reviewed the medical reports of imaging techniques in this study, but the images were not reviewed, which may have led to a deviation in the results. Therefore, we studied the patients in our center over the last 4 years, and the equipment and doctors remained unchanged during this period to reduce the deviation as much as possible.

In summary, our study evaluated the sensitivity of MG, US, MRI and combinations of these imaging modalities for the detection of small (≤2 cm) breast cancer. In this study, we found that MG has limited diagnostic sensitivity in patients with small breast cancer, especially those with dense breast tissue, and that US is better than MG at detecting small breast cancer, regardless of breast density. The combination of conventional imaging (MG and US) provided great accuracy in detecting small breast cancer and should be recommended first for small breast cancer. However, our study showed that further addition of MRI to MG and US could effectively improve the sensitivity and did not increase the mastectomy rate in small breast cancer. The evidence provided in this study suggests performing MRI routinely on the basis of MG and US for small (≤2 cm) breast cancer.

## Author contributions

**Conceptualization:** Hailong Chen.

**Data curation:** Hailong Chen, Jiaoqun Zhou, Qiang Chen.

**Formal analysis:** Hailong Chen, Jiaoqun Zhou, Yongchuan Deng.

**Investigation:** Hailong Chen, Jiaoqun Zhou, Qiang Chen.

**Methodology:** Hailong Chen, Jiaoqun Zhou.

**Supervision:** Yongchuan Deng.

**Validation:** Jiaoqun Zhou, Qiang Chen.

**Writing – original draft:** Hailong Chen, Qiang Chen.

**Writing – review & editing:** Yongchuan Deng.

## Supplementary Material

Supplemental Digital Content
